# A Novel *Anelloviridae* Species Detected in *Tadarida brasiliensis* Bats: First Sequence of a Chiropteran *Anellovirus*

**DOI:** 10.1128/genomeA.01028-14

**Published:** 2014-10-30

**Authors:** Samuel Paulo Cibulski, Thais Fumaco Teixeira, Francisco Esmaile de Sales Lima, Helton Fernandes do Santos, Ana Claudia Franco, Paulo Michel Roehe

**Affiliations:** a Virology Laboratory, Department of Microbiology, Immunology and Parasitology, Institute of Basic Health Sciences, Federal University of Rio Grande do Sul (UFRGS), Porto Alegre, Rio Grande do Sul, Brazil; b Fepagro Animal Health Institute of Veterinary Research Desidério Finamor (IPVDF), Eldorado do Sul, Brazil

## Abstract

Using metagenomic approaches, we identified a novel Torque teno virus from Brazilian free-tailed bats (*Tadarida brasiliensis*) (TT-TbV). The TT-TbV genome and deduced protein sequences share extremely low identity with known anelloviruses. Due to a high degree of phylogenetic divergence, such putative virus could not be allocated into any *Anelloviridae* genera.

## GENOME ANNOUNCEMENT

Torque teno viruses (TTVs) are small, nonenveloped viruses that contain a circular single-stranded DNA genome of negative polarity, currently classified within the family *Anelloviridae* (1). TTVs have also been identified in a number of species, including nonhuman primates, tupayas, cats, dogs, pigs, sea lions, and mosquitoes (2). Bats (order *Chiroptera*) have increasingly been recognized as sources of viruses that can potentially cause disease in humans and animals. Among these, a number of RNA and DNA viruses have been detected in bats, some of which have clear zoonotic potential, highlighting the importance of bat species as reservoirs for such agents (3). In this study, organs from 12 healthy Brazilian free-tailed bats (*Tadarida brasiliensis*) were submitted to our laboratory (IPVDF) as part of a continuous rabies surveillance program in Southern Brazil. The samples were pooled, macerated, filtered for removal of cell debris, and subjected to ultracentrifugation to concentrate the viral population. The DNA obtained was sequenced using the Illumina MiSeq system. The genome of a circular DNA virus of 2,367 nucleotides (nt), tentatively named TT-TbV, was identified. The genome organization of TT-TbV is similar to other members of *Anelloviridae*, with three open-reading frames (ORFs), unidirectionally transcribed and separated by an intergenic region of 556 nt. This intergenic region contains a 104-nt-long GC-rich sequence (74% GC content), forming three potential stem-loop structures, commonly found in the ssDNA virus. The ORF1 of 1,644 nt, responsible for encoding the putative capsid protein (Cap) of 547 amino acids (aa), has a low amino acid sequence identity (26%) in comparison to pine marten TTV1 (AEW87510). The N-terminus of ORF1 contains an arginine-rich region, with 21 arginine residues along the first 50 amino acids (42%), which is common in single-stranded DNA animal viruses (4). ORF2 encodes 99 amino acids and shares a 32% aa sequence identity with human Torque teno mini virus 2 (TTMV2, YP_003587883). TT-TbV ORF2 possesses the motif W-X_7_-H-X_3_-C-X_1_-C-X_5_-H, which is conserved among TTVs, TTMVs, and TTMDVs (1). ORF3 encodes 132 aa and shares an aa sequence identity of only 33% with the human TTV ORF3 (GenBank accession number BAB69914). Phylogenetically, TT-TbV forms a unique clade within *Anelloviridae*, indicating that TT-TbV is a putative novel *Anellovirus* species. Further studies should be conducted in the future to examine the potential biological role of TT-TbV in other bat species.

### Nucleotide sequence accession number.

The complete genome of TT-TbV has been deposited at GenBank under the accession number KM434181.

## References

[1] Okamoto H Mayumi M. 2000. Molecular virology of TT virus (TTV). Uirusu 50:259–271. (In Japanese.) 10.2222/jsv.50.259 11276815

[2] Okamoto H Takahashi M Nishizawa T Tawara A Fukai K Muramatsu U Naito Y Yoshikawa A. 2002. Genomic characterization of TT viruses (TTVs) in pigs, cats and dogs and their relatedness with species-specific TTVs in primates and tupaias. J. Gen. Virol. 83:1291–1297 12029143 10.1099/0022-1317-83-6-1291

[3] Lima FE Cibulski SP Elesbao F Carnieli Junior P Batista HB Roehe PM Franco AC. 2013. First detection of adenovirus in the vampire bat (*Desmodus rotundus*) in Brazil. Virus Genes 47:378–381. 10.1007/s11262-013-0947-6 23828618 PMC7088603

[4] Ng TF Manire C Borrowman K Langer T Ehrhart L Breitbart M. 2009. Discovery of a novel single-stranded DNA virus from a sea turtle fibropapilloma by using viral metagenomics. J. Virol. 83:2500–2509. 10.1128/JVI.01946-08 19116258 PMC2648252

